# Leukotoxins

**DOI:** 10.3390/toxins12040231

**Published:** 2020-04-07

**Authors:** Subramaniam Srikumaran

**Affiliations:** Department of Veterinary Microbiology and Pathology, College of Veterinary Medicine, Washington State University, Pullman, WA 99164-7040, USA; ssrikumaran@wsu.edu

Leukotoxins are the critical virulence factors of several Gram-positive and Gram-negative bacteria. Leukotoxin-deletion mutants exhibit drastically reduced virulence emphasizing the importance of these toxins as virulence factors in the pathogenesis of these bacteria. By targeting the leukocytes, they impair the ability of the hosts to mount an effective immune response. Some of them have hemolytic activity as well, which enables them to extract iron for bacterial growth.

The first group of articles deals with the leukotoxins produced by the Gram-positive bacterium *Staphylococcus aureus* which is an important pathogen that causes disease in both humans and animals. In humans, the disorders caused by *S. aureus* include life-threatening bacteremia, pneumonia, osteomyelitis, and infection of surgical sites. The management of these conditions has become more difficult with the emergence of methicillin-resistant *S. aureus* strains which are responsible for over 12,000 deaths in the US alone. Elucidation of the molecular mechanisms of action of the leukotoxins is critical for understanding the pathogenetic mechanisms of *S. aureus*, which in turn is essential for development of control measures against this pathogen. Venkatasubramaniam et al., [1] analyzed the expression of cytolytic pore-forming toxins of *S. aureus* which is regulated by the two-component sensing systems Sae and Agr. Here they report an important role of a leukotoxin component HlgA in the Newman strain to kill human red blood cells independent of Agr but dependent on a point mutation in SaeS. Liu et al., [2] investigated another *S. aureus* leukotoxin, the Panton–Valentine leucocidin, in an ex vivo model, and report that this leukotoxin colocalized with retinal neurons and induced glial and microglial activation, together with amacrine and microglial cells apoptosis, in a concentration- and time-dependent manner. Jeannoel et al., [3] studied the synergistic effects of influenza virus and *S. aureus* in a monocytic cell-line model, and report that influenza virus potentiates the pro-inflammatory action of heat-killed *S. aureus* and contributes to the cytotoxicity of alpha-hemolysin on monocytes, but only few synergistic interactions were relevant in the ex vivo model. As regards the *S. aureus* infection in animals, Hoekstra et al., [4] report that production of the bi-component leucocidin LukMF’ at higher levels is associated with clinical mastitis in cattle.

Nice et al., [5] investigated the leukotoxin produced by *Aggregatibacter actinomycetemcomitans*, a Gram-negative bacterium which is associated with localized aggressive periodontitis. They report that a significant fraction of the secreted LtxA exists in an outer membrane vesicle (OMV)-associated form, and this OMV-associated form is trafficked to the host cells by a cholesterol- and receptor-independent mechanism in contrast to the mechanism by which free LtxA is delivered.

The last two papers deal with two Gram-negative bacteria, *Mannheimia haemolytica* and *Bibersteinia trehalosi*, that are responsible for secondary bacterial infection in bovine respiratory disease that costs over $1 billion to the US cattle industry alone. The leukotoxin produced by *M. haemolytica* is cytolytic to all subsets of leukocytes. Since a large number of leukotoxic *M. haemolytica* isolates are beta-hemolytic, *M. haemolytica* isolates that are positive in the relatively easy to perform beta-hemolysis assays have been broadly assumed to be leukotoxic as well. By examining the leukotoxic and beta-hemolytic activity of a large number of *M. haemolytica* isolates, Bavananthasivam et al., [6] report here that beta-hemolysis may not be a reliable indicator of leukotoxicity of *M. haemolytica* isolates, and that cytotoxicity assays should be performed on *M. haemolytica* isolates to confirm their leukotoxicity. Since the leukotoxin is the critical virulence factor of *M. haemolytica* and *B. trehalosi*, characterization of leukotoxin epitopes is indispensable for developing vaccines that induce leukotoxin-neutralizing antibodies. While the leukotoxin of *M. haemoltica* has been well studied, characterization of *B. trehalosi* leukotoxin has lagged. By developing monoclonal antibodies against *B. trehalosi* leukotoxin, Murugananthan et al., [7] report that *B. trehalosi* leukotoxin contains a unique neutralizing epitope and a non-neutralizing epitope shared with *M. haemolytica* leukotoxin.
